# Carotenoid coloration and health status of urban Eurasian kestrels (*Falco tinnunculus*)

**DOI:** 10.1371/journal.pone.0191956

**Published:** 2018-02-08

**Authors:** Petra Sumasgutner, Marius Adrion, Anita Gamauf

**Affiliations:** 1 Department of Integrative Zoology, University of Vienna, Vienna, Austria; 2 Museum of Natural History Vienna, Vienna, Austria; 3 FitzPatrick Institute of African Ornithology, DST-NRF Centre of Excellence, University of Cape Town, Cape Town, South Africa; Justus Liebig Universitat Giessen, GERMANY

## Abstract

As the world experiences rapid urban expansion, natural landscapes are being transformed into cities at an alarming rate. Consequently, urbanization is identified as one of the biggest environmental challenges of our time, yet we lack a clear understanding of how urbanization affects free-living organisms. Urbanization leads to habitat fragmentation and increased impervious surfaces affecting for example availability and quality of food. Urbanization is also associated with increased pollution levels that can affect organisms directly, via ecophysiological constraints and indirectly by disrupting trophic interactions in multi-species networks. Birds are highly mobile, while an individual is not necessarily exposed to urban stressors around the clock, but nestlings of altricial birds are. Such a city-dwelling species with a long nestling phase is the Eurasian kestrel (*Falco tinnunculus*) in Vienna, Austria, which forage on a diverse diet differing in composition from rural habitats. Furthermore, prey items vary in nutritional value and contents of micronutrients like carotenoids, which might impact the nestlings’ health. Carotenoids are pigments that are incorporated into integument tissues but also have antioxidant and immunostimulatory capacity, resulting in a trade-off between these functions. In nestlings these pigments function in parent-offspring communication or sibling competition by advertising an individual’s physical or physiological condition. Anthropogenic disturbance and pollutants could have disruptive effects on the coloration of these traits. In this study, we measured carotenoid based coloration and other indicators of individual health (body condition and susceptibility to the ectoparasite *Carnus hemapterus*) of 154 nestling kestrels (n = 91 nests) along an urban gradient from 2010 to 2015. We found skin yellowness of nestlings from nest-sites in the city-center to be least pronounced. This result might indicate that inner-city nestlings are strongly affected by urban stressors and depleted their stores of dietary carotenoids for health-related functions rather than coloration. In addition, skin yellowness intensified with age and was stronger pronounced in earlier nests. Since the immune system of nestlings is still developing, younger chicks might need more antioxidants to combat environmental stress. Additionally, parasite infection intensity was highest in nestlings with less intense skin yellowness (paler or less yellow pigmented integuments) and in earlier nests of the season. In combination with results from previous studies, our findings provide further support for the low quality of the inner-city habitat, both in terms of productivity and individual health.

## Introduction

Wildlife around the globe faces the dangers of a novel, quickly spreading habitat infiltrating the natural environment: urban areas, i.e. cities and other human settlements. Through the drastic changes imposed by cities, such as increased impervious surfaces, habitat loss and fragmentation, noise, light and chemical pollution, introduced alien species and predators, and diet alteration, urbanization acts as a filter for species communities [1–3]. This leads to biodiversity loss through both random processes and mal-adaption of some species [4]. Thus, urbanization is a novel and strong evolutionary force [5, 6]. In avian research, species are categorized according to their adaptability to urban landscapes in urban avoiders, adapters or exploiters [1, 7], reflecting their ability to cope with this novel habitat. Some species seem to cope well with urban environments and react with rapid evolutionary adaptations to city life [8–10]. Other species, however, show negative responses to anthropogenic stressors in terms of productivity and individual health. Furthermore, urbanization influences host susceptibility to parasites and infectious diseases and can intensify the detrimental effects of pathogens by weakening the immune system [11].

Birds are highly mobile, but the nestlings of altricial species might be especially exposed to urban landscapes since they are bound to their nest and fully dependent on parental care for most of their early development. Thus, they must endure variation in food availability, sibling competition, exposure to parasites and predators, adverse weather and other environmental stressors, with some buffer by their parents [12].

Raptors are particularly interesting to study in an urban context. As umbrella species, their loss can have cascading effects on ecosystem levels [13–15]. Yet, raptors depend on large suitable habitats with stable prey populations and might be vulnerable in urban areas [16]. In spite of this, the trend of raptors colonizing cities is on the rise, with more species moving into urbanized areas, for example American kestrel (*Falco sparverius*, [17]), Black sparrowhawk (*Accipiter melanoleucus*, [18]), Cooper’s hawk (*Accipiter cooperii* [19]), Crowned eagle (*Stephanoaetus coronatus*, [20, 21]), Mississippi kite (*Ictinia mississippiensis*, [22]), Merlin (*Falco columbiarus*, [23]), Northern goshawk (*Accipiter gentilis*, [24]), Peregrine falcon (*Falco peregrinus*, [25]), and the focus of this study, the Eurasian kestrel (*Falco tinnunculus*), or hereafter simply kestrel. Kestrels breed in many European cities (e.g. [26–33]), and the diet of urban kestrels can heavily differ from their rural counterparts [34]. In Vienna, kestrels breeding in highly urbanized areas increasingly feed on birds, reptiles and insects compared to kestrels breeding in the outskirts of the city, which primarily feed on voles [35, 36]. For raptors living in urban landscapes, the altered availability of prey species might be challenging, but could also be beneficial specifically for raptors that feed on avian prey or have smaller home range sizes [37]. However, cities may pose subtler physiological risks to raptors, even to those species which appear to do well in urban areas in terms of number of breeding pairs. Examining the mechanisms behind the perceived success of raptors in urban areas can thus help to establish whether these species might be affected by an ecological trap mechanism or whether they are true urban-adapters.

Stress leads to higher glucocorticoid levels; prolonged stress might result in lower body condition [38–42]. To cope with a stressful environment and prevent cell damage, living organisms need to produce or ingest molecules with antioxidant properties. One class of antioxidants are carotenoids [43], yellow to red pigments which are strictly dietary for vertebrates [44, 45]. The total amount of carotenoid pigments available for an individual will depend on the quantity and the quality, in terms of carotenoid content, of the ingested food [46–48]. Raptors feed on a variety of prey species that might thus differ in their carotenoid content, with birds having higher carotenoid content than mammals [49]. Kestrels might face a trade-off to meet their dietary needs: voles (*Microtus spp*.) are potentially more efficient to hunt [34], if sufficiently available, and have high calorie but low carotenoid content, whereas their alternative prey (small birds or larger insects) might be more difficult to catch for a specialized vole hunter, and is comparably less calorific, but higher in its carotenoid content [50, 51]. Carotenoids serve many functions in animals, most visibly the coloration of nearly all vertebrate integumentary tissues like skin, eyes, reptilian scales, bird feathers and beaks [52], but also other physiological functions, e.g. antioxidant capacity and immunomodulatory properties [44, 53–55]; but see [43, 56, 57]. Due to the diverse functions of carotenoids, trade-offs in the allocation of these important micronutrients have been proposed [51]. Carotenoid-based coloration of birds’ bare skin and beaks has been identified as a dynamic, condition-dependent trait [58] in adults [59] and nestlings [49, 60], including kestrels. Additionally, a strong correlation between carotenoid-based integument coloration and circulating carotenoids is known [49]. This suggests that the expression of the trait is an honest indicator of individual quality [61, 62] and thus play a key role in social communication [63–65].

Especially nestlings need carotenoids, since their immune system is not yet fully developed, and hatching asynchrony or insufficient parental care can lead to undernourishment [66–68]. It is known from other raptor species that the intake of carotenoid-rich food sources can improve individual health, specifically during the nestling phase [63, 69, 70]. Additionally, these traits function in parent-offspring communication or sibling competition by advertising an individual’s physical or physiological condition [71, 72]. In this regard, anthropogenic disturbance or environmental pollutants could have disruptive effects on the coloration of these traits [49, 73], thereby interfering with communication processes.

Living in urban environments, the exposure to anthropogenic stressors might not only lead to immediate health problems, like lower body conditions of adults [74, 75] and juveniles [76], but also to reduced reproductive performance and survival (review in [77]). The change in individual densities probably also alters the interactions between hosts and pathogens. This can lead to more abundant parasites or vectors and thereby facilitates the rise of pathogens and multiplies the transmission of infectious diseases in urban environments [78, 79]. Together with increased physiological stress, the severity of diseases might increase, including higher infection prevalence with haemo-, endo-, and ecto-parasites [80]. The parasitic fly *Carnus hemapterus* is a common ectoparasite of many bird species, including kestrels [30, 81]. They mainly infest nestlings, with the highest infection rates in last-hatched chicks, i.e. junior siblings [82]. Infections with parasites have been connected to reduced expression of carotenoid-based signals, reflecting the strong immune response against parasites [54, 83].

We propose two competing hypotheses regarding integument coloration: (i) dietary antioxidants are supposedly restricted in vole hunters; since kestrels in the city center increasingly feed on alternative prey (mainly insectivores), inner-city nestlings should show more intense carotenoid (i.e. yellow) coloration or alternatively, (ii) because environmental stress is increased in the city, carotenoids are used for antioxidant defense rather than coloration why inner-city nestlings should show less intense carotenoid coloration despite their potential higher availability.

At the same time breeding performance is known to be lower in inner-city kestrel pairs and malnutrition was named as one of the main factors leading to the high nestling mortality [34,79]. Hence, high physiological stress could lead to a measurable drop in health-related traits. On the other hand, already smaller clutches and lower fledged brood size could result in reduced sibling competition for limited food, resulting in an overall higher quality of surviving fledglings (along the offspring size-number trade-off; [80]). Both of these competing hypotheses predict a significant correlation between the urban gradient and individual nestlings’ health, measured as (1) carotenoid-based integument coloration, (2) body condition, and (3) susceptibility to the ectoparasite *Carnus hemapterus*.

## Material and methods

### Study system

**Fig 1:**
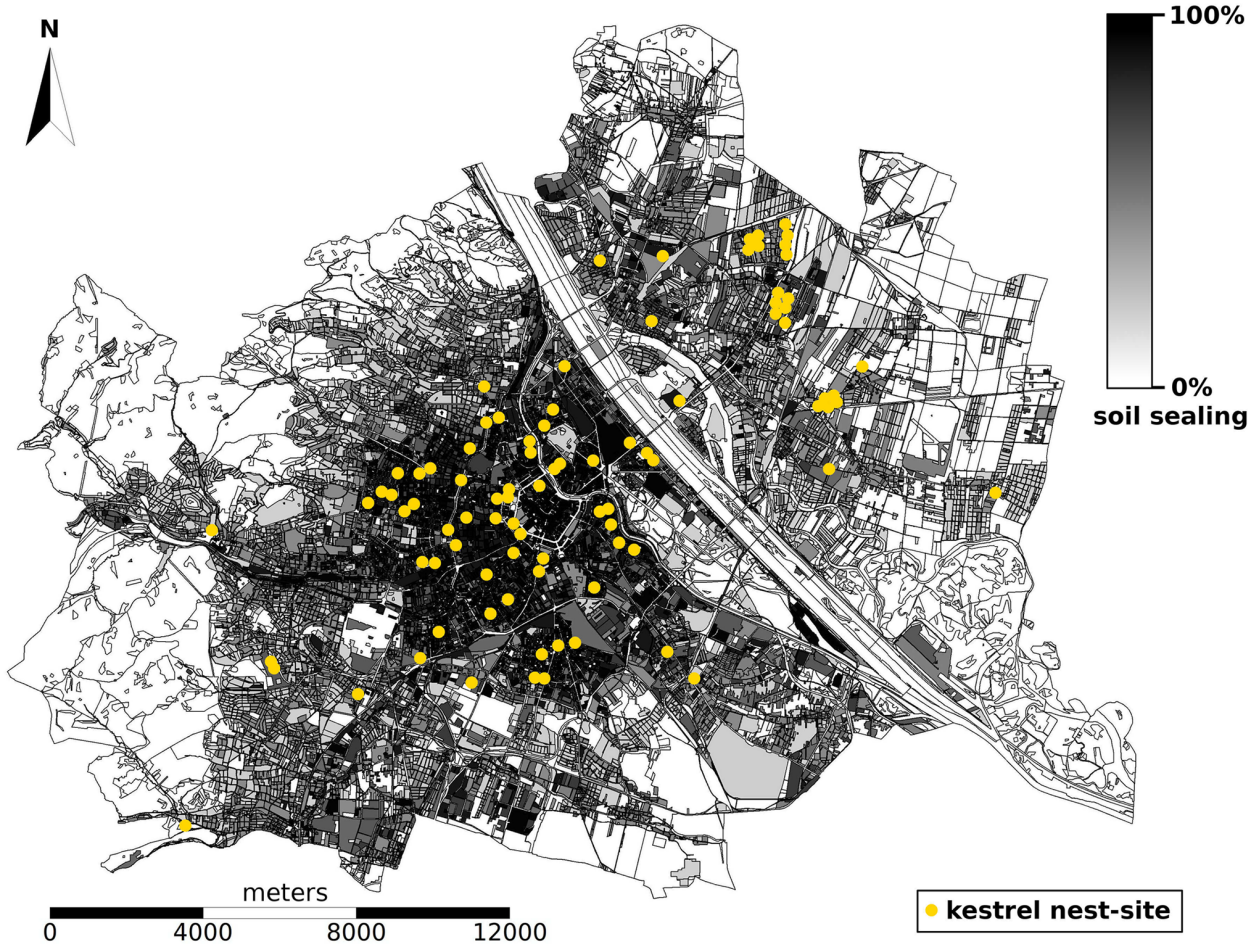
Urban study area (243 km^2^) in Vienna, Austria. The urban gradient is displayed from black to light grey (white areas (<1%) are outside the study area), and locations of kestrel nest-sites are indicated in yellow.

The Eurasian kestrel is one of the most common birds of prey of the Palearctic region. It is widespread throughout its range, occupying a wide range of open natural habitats to human modified landscapes [26, 34]. In Vienna, kestrels are the most common aerial predators, reaching densities of 89–122 breeding pairs/100 km^2^ [32], which is higher than in other European cities or rural areas [28, 84]. Kestrels are specialized in hunting voles, but also feed on other small vertebrates and large invertebrates [35, 36, 85]. All data for this study were gathered in the city of Vienna, Austria (48°12’N, 16°22’E; 415 km^2^; 150–500 m a.s.l.; 1.8 million inhabitants). We defined the urban gradient by the percentage of sealed soil and excluded rural areas with <1% sealed soil, thus comprising an urban study area of 243 km^2^ (Fig 1). The proportion of impervious surfaces was calculated using ESRI ArcGIS 10 based on coverage by buildings and traffic areas for r = 500 m around the nest [32, 33]. Kestrel nesting sites used in this study were found from 19% soil sealing in suburban areas to 97% in the inner city.

### Field procedure: Morphometric measurement and ectoparasite counts

Kestrel nesting sites in the city of Vienna were located by sight during systematic search and with the help of the general public from 2010 to 2015 and regularly checked for occupation during the nesting season [32, 33, 35, 36]. For this study, we used data from 91 kestrel nest-sites from all six years. When occupied, nests were visited two to three times if accessible through the attic, by tree and façade climbing or with help from the Viennese fire-fighters to assess the number of eggs, number of hatched nestlings and number of fledged chicks. The nestlings were ringed, weighed and measured: length of the fourth primary (numbered from the outermost towards the inside, and length of the first rectrix to the nearest 0.1 mm (with calipers) and wing length to the nearest mm (with a zero-stop ruler; [86]; n = 556 nestlings). One randomly chosen nestling per brood was screened for ectoparasites (n = 154). The most significant arthropod ectoparasite in kestrels is the blood-sucking fly *Carnus hemapterus* [87]. Parasite infestation decreases with age of the nestlings, and the youngest chick of a clutch usually has most parasites [82, 88]. The number of all ectoparasites was counted in each nestling on the day they were ringed (aged six to 33 days, supplementary material S1 Fig for age distribution of sampled kestrel nestlings), and parasite load was defined as an ordinal score (see [89] for a similar approach): 0 (no *C*. *hemapterus* visible), 1 (one to three *C*. *hemapterus*), 2 (four to nine *C*. *hemapterus*), 3 (more than 10 *C*. *hemapterus* on the nestling’s skin). Blood from nestlings was collected for genetic sex determination, by puncturing the brachial vein with a sterilized 21 Gauge needle and extraction of the blood through capillary action with 75μl heparinized capillary tubes after disinfection of the skin with alcohol swabs. In the following lab procedure we extracted DNA from full blood using the standard protocol of QIAGEN DNeasy Blood & Tissue kit. PCR products of the sex specific primers 2718R and 2550F [90] were visualized on 2% agarose gels. See details in [33].

### Color measurement

Similarly to the ectoparasite count was skin yellowness measured in one randomly chosen nestling per brood. This resulted in a reduced dataset for both variables of n = 154 nestlings from 6 breeding seasons and 91 different nest locations. The colors of the unfeathered skin covering tarsus, cere and eye ring (orbital ring) were scored by comparing the bird under natural light conditions to a reference color charts booklet [91]. Scoring colors by visual matching with color charts has been widely used in studies on avian coloration [49, 50, 63, 69, 70, 83, 92–94]. The color chart method was preferred to other methods, such as spectrophotometry, because of fieldwork constrains and because it is better suited to quantify the coloration of irregular surfaces such as ceres and tarsi [95]. However, to validate our method we compared the results using color charts with spectrophotometry in one study year on a smaller sample size (see color validation study below). Since there were no colorimetrics available for the color charts used in this study, we used an image scanner (Canon imageRUNNER Advance C5235i) to extract high resolution digital photographs of the color charts in standardized lighting conditions. The images of the color charts were then analysed with GIMP [96]. For each color chart we measured the average red, green and blue components (RGB color space). The three components have equal values in white (high values) and black (zero values) colors, respectively. The relative difference of the three-color components determines the hue of the color. For example, the bright and saturated yellows have high values in the red and green component and very low values in the blue component. We calculated a “yellowness score” for each color chart by subtracting the ratio of red and green values from the blue value (essentially using the blue value, correcting for red and green; [63, 83, 93, 97]). We used this RGB-derived color chart score as a continuous variable in subsequent analyses [95, 98, 99] calling it “skin yellowness” hereafter.

Since cere skin yellowness and skin yellowness of the orbital ring were highly correlated (cor = 0.93; p < 0.001), but tarsus skin yellowness not as strongly, we ran a Principal Component Analysis (PCA) on the measurements. The PCA resulted in two principal components, explaining 97.3% of the variation in the data, with PC1 consisting mainly of cere color and eye ring color and PC2 consisting mainly of tarsus color (S1 Table). PC1 was used as “face skin yellowness” and PC2 as “tarsus skin yellowness” in subsequent analyses. Note that the values of PC1 and PC2 are antipodal: low values of PC1 represent more intense yellow colors and vice versa, low values of PC2 represent dull/whitish shades of yellow.

### Spectral reflectance with spectrophotometry

To estimate the reliability of the color chart method used in this study, photospectrometral color measurement was carried out in the field season of 2015, measuring both tarsus and cere coloration of 25 kestrel nestlings. Measurements were all taken by the same person (MA) using a portable JAZ photospectrometer (Ocean Optics, Inc.) with a pulsed xenon light source (190–1000 nm optical range) and an optical fiber with a self-made probe pointer, firmly attached to the optical fiber. The handheld probe pointer was cut in a 45° angle and could then be placed firmly on the animals’ skin (coincident oblique technique; [100]) to avoid distortions of the measurements due to the glossy surface of the skin. Reflectance of each body patch was measured three times, removing the probe pointer in between measurements. The raw data files were analyzed with the package pavo [101].

To compare the two different color measurements, color charts and spectral reflectance, we extracted three colorimetric from the reflectance curves within the 400–700 nm spectral range: (a) carotenoid chroma (the difference between the reflectance at 700 nm and 450 nm divided by the reflectance at 450 nm, i.e. relative reflectance in the region of highest reflectance in carotenoid-based colors), a measure of carotenoid concentration [100], (b) carotenoid saturation (after [94, 95]), the ratio between total brightness (area under the curve) and the reflectance in the region of interest (from 550 nm to 700 nm, corresponding to yellow, orange and red wavelengths [95]); and (c) spectral saturation of the yellow segment (S1y), calculated as the sum of the reflectance values in the wavelength range 550–625 nm (associated with yellow colors) divided by the total reflectance [95, 101].

### Age determination and lay date

Age of the nestlings was determined from plumage development, as the exact age (in days) was seldom known from direct observation [26]. Another method, developed by BirdLife Finland (http://netti.nic.fi/~mattisj/petopull_ika_index.html), estimates the age of nestlings only from wing length. These two methods resulted in differing nestling ages, the finish formula making the Viennese nestlings on average 2.36 ± 0.06 (standard error) days younger than the method by [26], which is based on kestrel populations in Germany. Since both methods for age determination are based on European kestrel populations outside our study area, the average of both values was used in statistical analyses.

If not known from direct observation, the laying date of the first egg of the clutch was inferred by subtracting 30 days, the average breeding time of kestrels (median 29 days [102]; about 30 days [26]) from the hatching day, calculated from the age of the nestling at the time of ringing. The lay date is used as Julian day throughout (1 = Jan 1). To correct for the size difference of nestlings due to hatching asynchrony [103–105], we ranked the nestlings within the brood as senior (“1”, first-hatched/largest, 1–3 chicks per brood) sibling, junior (“3”, last-hatched/smallest) sibling and “2”, all the other nestlings in between.

### Statistical analysis

To validate the color chart measurements with spectral reflectance values we used Pearson’s correlation and report the according r and p-values. Color chart measurements were done every year by a different person (2010–2012 PS, 2013 Tomislav Gaspar, 2014 Anna Kreiderits, 2015 MA). To ensure no observer bias we run another validation by fitting “year” as a fixed effect—with no significant relationship: χ^2^_(n = 154, df = 5)_ = 8.04, p = 0.154. However, the year is fitted as random term throughout to account for a potential year/observer effect.

Nestlings were not all measured at the same age; hence, to model body mass as response variable we controlled for their age by fitting the wing length (linear and quadratic since body mass gain declines before nestlings fledge [106]) together with other predictor variables we were interested in (see below). However, when fitting body condition as explanatory variable, we used residuals to fulfil the assumption of non-correlated fixed effects following the method described by Roulin et al. [106]: First we extracted residuals from a second-order curve of body mass on wing length (with each kestrel nestling appearing only once in the analysis, n = 531), and then removed variation in these residuals explained by sex (two-way ANOVA; females are heavier than males). The latter residuals were our body condition index used as fixed-effect in statistical analyses (see [49] for a similar approach).

Each of the four health parameters used in this study (body condition index, ectoparasite infection, face skin yellowness and tarsus skin yellowness) was used as a response variable in generalized linear mixed models (GLMM) with age, sex, rank of the nestlings and the egg-laying date as explanatory variables, together with the urban gradient as additive factor and in interaction with the respective other health measurements. A global model was fitted with non-correlated explanatory variables, where all quantitative variables (including the response if applicable) were centred and scaled to ensure that effect sizes were on a comparable scale [107]. A model set was generated using the Anova (type = “III”) function (car package [108]), and candidate models were ranked in relation to each other using AICc values [109]. The GLMMs for body mass, face skin yellowness and tarsus skin yellowness followed a Gaussian error structure. Ectoparasite infection was analyzed as count data with Poisson. The year (breeding season) and nest ID (location where the nestlings were sampled) were included as random terms, to account for pseudoreplication due to the lack of independence of several individuals produced within years or the same nest between different years [110]. All analyses were conducted in R [111] using Rstudio [112] and the lme4 [113], car [108], MASS [114], MuMIn [115], effects [116, 117], lattice [118] and RVAideMemoire [119] packages. Post-hoc comparisons between factor variables were performed using the package lsmeans [120]. Pseudo-R^2^ for GLMMs was calculated after [121] using the function “r.squaredGLMM” implemented in the package MuMIn, for GLMMs with Gaussian and Poisson error distributions.

### Ethical statement

The study was performed under license from the Environmental Protection Bureau of Vienna (MA22/1263/2010/3), the Ministry for Science and Research (BM.WF– 66.006/0021-II/3b/2013) and the ethics committee of the University of Veterinary Medicine, Vienna (BGBI.Nr.501/1989i.d.g.F.). All data were acquired strictly following current Austrian and EU law as well as the Weatherall Report and the guidelines for treatment of animals in behavioural research and teaching (ASAB 2015).

## Results

### Color validation study

Photospectrometral color measurement was successfully carried out on 25 kestrel nestlings from 25 different nest sites in the breeding season of 2015. Correlations of kestrel nestlings’ skin yellowness from visual classification using reference color charts (RGB) and photospectrometral color measurements were strongly correlated using the birds’ tarsi: S1y (Pearson’s product-moment correlation: r = -0.81, p < 0.001), carotenoid chroma (r = 0.73, p < 0.001) and carotenoid saturation (r = -0.72, p < 0.001); and the birds’ ceres: S1y (r = -0.63, p < 0.001), carotenoid chroma (r = 0.41, p = 0.043) and carotenoid saturation (r = -0.43, p = 0.033); and when combining both: S1y (r = -0.69, p < 0.001), carotenoid chroma (r = 0.48, p < 0.001) and carotenoid saturation (r = -0.32, p = 0.029).

### Carotenoid coloration and health status

**Fig 2:**
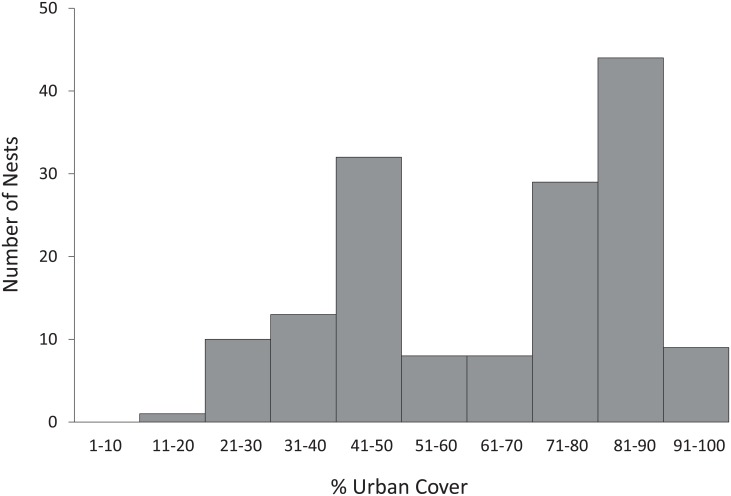
Frequency plot showing the distribution of kestrel nest sites (N = 154) along the urban gradient in 10% slots.

Kestrel nesting sites were distributed across the city within a wide range of degree of urbanization (19.4–96.8%, Fig 2). We found no significant relationship between the degree of urbanization around kestrels’ nesting sites and the body mass of nestlings Body mass could not be explained by any other explanatory variables than wing length and sex (Table 1, supplementary material S2a and S2b Fig).

**Table 1:** Results of best models explaining health parameters (body mass, ectoparasite infection intensity, tarsus skin yellowness, face skin yellowness) of urban kestrel nestlings. Significant values in bold (n = 154 individuals, 91 different nest sites between 2010–2015).

Response variable	Estimate	SE	Chi^2^	Df	P(>Chi^2^)	R^2^ for GLMM	AICc
**Body mass**						54.13	355.90
(Intercept)	0.12	0.10			0.224		
Wing length	0.74	0.06	147.41	1	**<0.001**		
Wing length^2	-0.30	0.04	44.90	1	**<0.001**		
Sex ^a^	0.31	0.12	7.20	1	**0.007**		
**Ecto-parasite infection**						12.86	243.60
(Intercept)	-1.25	0.26			**<0.001**		
Laying date	-0.37	0.15	5.80	1	**0.016**		
Face skin yellowness	0.40	0.15	6.62	1	**0.010**		
**Tarsus skin yellowness**							228.60
(Intercept)	-1.89	5.05	-0.38		0.708		
**Face skin yellowness**						24.05	226.60
(Intercept)	-0.21	0.13			0.101		
Sex ^a^	0.34	0.15	5.03	1	**0.025**		
Age	-0.26	0.08	11.56	1	**<0.001**		
Laying date	0.13	0.08	2.93	1	0.087		
Urban gradient	0.19	0.08	6.10	1	**0.014**		

^a^: Sex(male) was the reference category.

**Fig 3:**
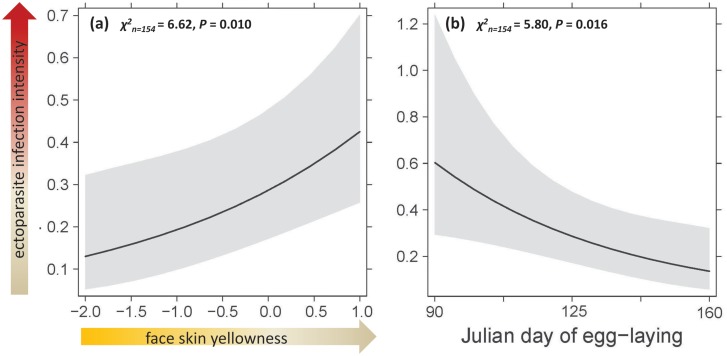
Result of the best model for ectoparasite infection intensity of urban kestrel nestlings. GLMM effect sizes of (a) face skin yellowness and (b) the egg-laying date. The model explains 13% of the variance in ectoparasite infection intensity.

Ectoparasites *Carnus hemapterus* were found on 41 of 154 nestlings (26.6% infection prevalence; 154 broods between 2010–2016 at 91 different nest sites), the infection intensity ranged from one to 50 individuals of *C*. *hemapterus* per kestrel nestling (mean = 1.4±0.09SE). Infection intensity with *C*. *hemapterus* was not directly related to the urban gradient (the most parsimonious model did not include the urban gradient or any of the interaction terms considered). Ectoparasite infection intensity was higher in nestlings with less intense face skin yellowness (GLMM ‘face skin yellowness’ term: χ^2^_(152)_ = 6.62, p = 0.01; Fig 3a) and also in nestlings hatching earlier in the breeding season (GLMM ‘egg-laying date’ term: χ^2^_(152)_ = 5.80, p = 0.016; Fig 3b). The model with face skin yellowness and laying date explained 13% of the variation in ectoparasite infection intensity.

**Fig 4:**
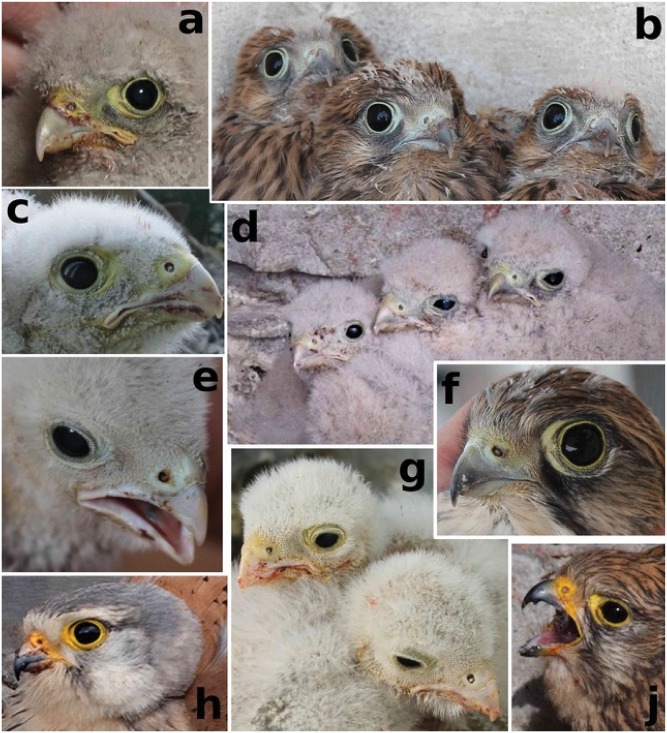
Photographs of kestrel (a-g) nestlings, (h) adult male and (j) adult female from Vienna, showing great variation in coloration of cere and eye ring skin. Color intensity usually increases from (d, e, g) young to (a, c) older chicks until (b. f) fledgling. Adults have very intensely yellow colored skin, specifically the males.

**Fig 5:**
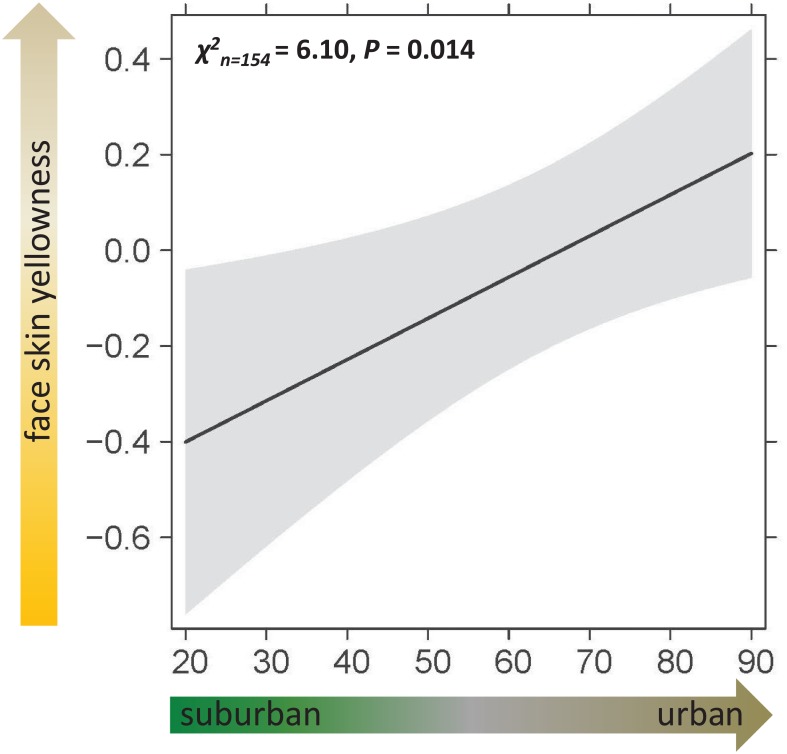
GLMM effect sizes of the urban gradient on face skin yellowness of kestrel nestlings from GLMM (see Table 1) fitted with sex, age and the laying date as additional co-variates (note: smaller values are more intense shades of yellow, and more suburban areas). The model explains 24% of the variance in face skin yellowness.

In terms of carotenoid based coloration (RGB-values from color chart classification; Fig 4), the most parsimonious model for tarsus skin yellowness was the null model (Table 1. Face skin yellowness intensity decreased with an increasing degree of urbanization (GLMM ‘urban gradient’ term: χ^2^_(149)_ = 6.10, p = 0.014; Fig 5), increased with age (GLMM ‘age’ term: χ^2^_(149)_ = 11.56, p<0.001; S3a Fig), and differed between sexes (GLMM ‘sex’ term: χ^2^_(149)_ = 5.03, p = 0.025; S3b Fig), with males being more intensely colored than females (post-hoc pairwise comparison, least-squares means: t-ratio = -2.20, p = 0.029). Additionally, nestlings from earlier broods showed slightly more intense face yellowness (GLMM ‘egg-laying date’ term: χ^2^_(149)_ = 2.93, p = 0.087, S3c Fig). The best model explained 24% of variation in face skin yellowness.

## Discussion

**Fig 6:**
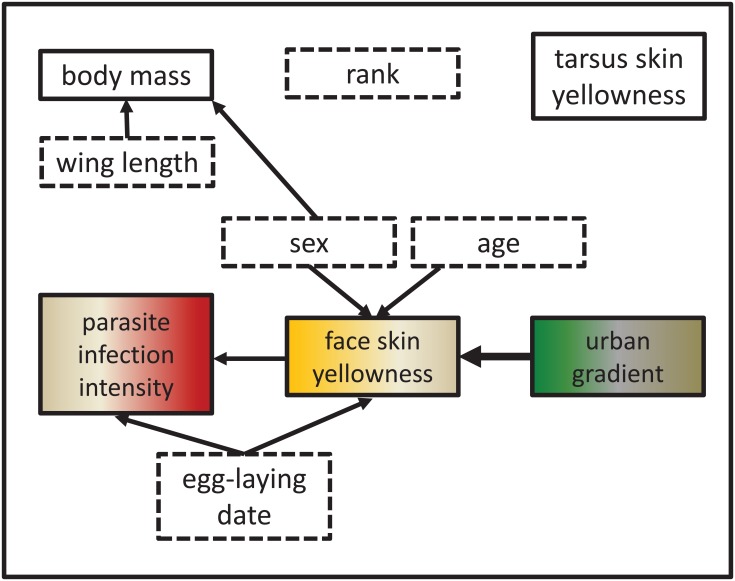
Schematic diagram showing interrelations of health-related variables in urban kestrel nestlings. Variables in solid-lined boxes have been used as response and explanatory variables in GLMMs, variables in dashed boxes only as explanatory variables. The arrow for the urban gradient is displayed in bold to underline its importance in the main hypotheses.

Along the urban gradient, we found skin yellowness of nestlings from nest-sites in the city-center to be least pronounced, indicating that they are stronger affected by novel urban stressors than nestlings from more rural areas, and indeed allocate these carotenoids as important micronutrients to antioxidant defense instead of coloration. In addition, skin yellowness intensified with age and was stronger pronounced in male nestlings. The other health indicators used, i.e. body condition and parasite infection intensity were not directly linked to the urban gradient, but one could argue some indirect effects (see Fig 6).

Carotenoid-based coloration of birds’ bare parts is a dynamic, condition-dependent signal, and supposedly an honest signal that reflects an individual’s health status [58, 60]. Previous findings in our system have shown that kestrels nesting in the inner-city (of Vienna and other European cities, e.g. [27, 28, 122] feed their chicks on a higher proportion of carotenoid-rich prey (i.e. birds, lizards and insects [64, 123, 124]); than their suburban conspecifics [32, 33, 35, 36]. Additionally, there is a known difference in brood sizes due to a high nestling mortality that might reflect a poorer health condition along the urban gradient [33]. Following these findings we hypothesized that either (i) the intensity of integument carotenoid coloration is highest in nestlings from more urbanized areas due to their carotenoid rich diet; or (ii) the opposite, since the exposure to urban stressors could result in a depletion of carotenoids for antioxidant defense. In line with hypothesis (ii), we found nestlings from the city center to have least intense yellow coloration, despite their supposedly higher carotenoid intake through their urban diet.

There are several non-exclusive explanations for this finding: (1) urban stressors, e.g. chemical, noise and light pollution, heavy metals and direct disturbance might result in oxidative stress [125], hence city kestrels need to use all dietary antioxidants available to reduce oxidative damage. Furthermore, inflammations and parasite infections might be more frequent in urbanized areas (see for example [126]), which further activates the immune system that requires more circulating carotenoids. Thus, carotenoids are allocated to physiological needs and cannot be used for integument pigmentation anymore [43]. (2) urbanization changes whole ecosystems and the species networks [127], thus, increased oxidative stress will act on all trophic levels from primary producers to apex consumers. For example, carotenoid content of urban prey species such as Great tits (*Parus major*) might be lower than that of rural prey [128], since urban passerines mainly forage on low-quality food resources themselves (i.e. caterpillars with low carotenoid contents, [129]), due to lower carotenoid levels in inner-city trees [130]. Thus, the entire urban food chain might be affected by a limited availability of dietary antioxidants and strong environmental stress. As a result, there might be an overall lower carotenoid content of biota in urban areas, just as evidence from plants [131] and passerines [132–135] does.

If explanation (1) would hold true and urban stressors indeed create a health challenge for city kestrel nestlings and all carotenoids are used for antioxidant defense, we would expect a direct link between coloration and parasite infection intensity. Indeed, we find higher infection intensities with the ectoparasite *Canus hemapterus* in nestlings with paler face skin yellowness. Since face skin yellowness also decreased with the degree of urbanization, the combination of these results would lead to the conclusion that nestlings from inner-city nest sites tend to have more ectoparasites. However, we did not find an interaction between coloration and the urban gradient explaining parasite infection intensity. We are confident that our result is not due to age, with less intensively yellow colored nestlings having more parasites because they might be younger (in line with the tasty chick hypothesis [82, 136]), since ectoparasite infection was not correlated with age, but mainly with yellowness. Finally, parasite infection intensity was also highest in nestlings from early nests, decreasing over the course of the breeding season. A seasonal trend of infection with ectoparasites is known from raptor studies, but usually parasite numbers increased during the season [106], although Sumasgutner et al. [88] found a seasonal decline of parasite infection intensity specifically in kestrel nestlings, limited to nest boxes that were left un-cleaned and consecutively used for breeding over several years. In Vienna, we basically have the same situation, since kestrels mainly breed in roof openings with pellets and other prey remains from earlier breeding attempts, where *C*. *hemapterus* can overwinter.

The potential link between carotenoid-coloration and the urban gradient described above was found in the facial integuments (i.e. cere and orbital ring) of kestrel chicks, but not for their tarsal skin. Cere and orbital ring color scores in our kestrel nestlings were often identical (62% of all 454 measurements) or closely related. Overall, there was less variation in tarsus color scores than in facial coloration scores (the legs being more intensely yellow whereas facial coloration ranged between greenish-yellow and bluish-yellow colors; see Fig 4). The intensity of yellow integument coloration of kestrel nestlings increased with age for the facial coloration (but not for the tarsal skin), as known from other raptor nestlings [63], including kestrels [68]. This positive age-dependence of carotenoid-based coloration might reflect the condition of the nestlings which usually increases with age and the maturation of the immune system. Furthermore, carotenoids had more time to accumulate in the integuments of older nestlings, and the absorption and deposition processes are more mature [68]. To account for this known correlation, we controlled for “age” by fitting it as additional co-variable throughout which seemed especially important since we measured the study broods in a rather large age window due to the limited accessibility of many nest sites. Additionally, in our study, male nestlings were more intensely colored (face skin yellowness) than females, which is in line with a study on Marsh harrier (*Circus aeruginosus*) nestlings [69], but not with other kestrel nestlings [68]. Possibly males and females have different carotenoid allocation strategies, resulting in differential maturation of traits important for sexual selection. This trait may already be present in nestlings but does not acquire its signaling function until adulthood [69]. Another possible explanation is that sexes follow different growth strategies. Females, the larger sex in size-dimorphic raptors [137], invest more in growth and require more carotenoids as antioxidants against growth-related free radical production [138]. This explanation would lead to less intense skin yellowness in females, as well as the possibility that female nestlings are larger and stronger and could therefore, to some extent, control the distribution of prey items among siblings. Possibly, they thus consume larger prey which is a poorer source of carotenoids (mammals) and leave the smaller prey (birds and insects, with higher carotenoid contents) to their brothers [70].

### Conclusion

The results from this study are further support for the hypothesis that kestrels breeding in Vienna don’t choose nest-sites according to the habitat quality and are somewhat unable to assess the true quality of the surrounding hunting territory [32, 33]. Together with our new the findings on carotenoid coloration as an indicator for individual health status, kestrels breeding in Vienna’s city center could indeed be in danger of falling into an ‘ecological trap’. All data used in this manuscript are available in the supporting information S1 Data file.

## Supporting information

S1 Table Results of a Principal Components Analysis (PCA) on the colour chart measurements of 3 body parts of kestrel nestlings: tarsus, cere and orbital ring. PCs 1 and 2 were used as "face skin yellowness" and "tarsus skin yellowness", respectively, throughout the manuscript.
(PDF)

S1 Fig Age distribution of kestrel nestlings considered for the analyses. We randomly selected one nestling per brood (n = 154 individuals, 154 broods, 91 different nest sites between 2010–2016) with an age range of six to 33 days.
(PDF)

S2 Fig Result of the best model of a GLMM on body mass of urban kestrel nestlings (see Table 1): Effects of (a) wing length, and (b) sex contribute significantly to the best model. The model explains 51% of the variance in body mass (note: inward ticks on the x-axis show sample sizes).
(PDF)

S3 Fig Result of the best model of a GLMM on face skin yellowness of urban kestrel nestlings (see Table 1). Effects of (a) age of nestlings, (b) nestling sex, (c) egg-laying date and urban gradient contribute significantly to the best model. The model explains 24% of the variance in face skin yellowness.
(PDF)

S1 Data All data used in this manuscript for 154 kestrel nestlings from 91 different nest sites collected between 2010–2016 including a comprehensive legend explaining the variables (details of nest site and location along the urban gradient, breeding stage, morphometric measurements, parasite infection and color assessment). (XLSX)
