# Supplementary material for: Carotenoid coloration and health status of urban Eurasian kestrels (Falco tinnunculus)
Source: PLoS One. 2018 Feb 8;13(2):e0191956. doi: 10.1371/journal.pone.0191956 (PMC5805255; doi:10.1371/journal.pone.0191956)
Supplement: S1 Table — PCs 1 and 2 were used as "face skin yellowness" and "tarsus skin yellowness", respectively, throughout the manuscript. (PDF) [file pone.0191956.s001.pdf]

Supporting information:

"Carotenoid coloration and health status of urban Eurasian kestrels (*Falco tinnunculus*)"

Petra Sumasgutner, Marius Adrion, Anita Gamauf

S1 Table: Results of a Principal Components Analysis (PCA) on the colour chart measurements of 3 body parts of kestrel nestlings: tarsus, cere and orbital ring. PCs 1 and 2 were used as "face skin yellowness" and "tarsus skin yellowness", respectively, throughout the manuscript.

**Rotation:**	**PC1**	**PC2**	**PC3**
Tarsus RGB	0.31	-0.95	0.07
Cere RGB	0.66	0.27	0.71
Orbital ring RGB	0.69	0.17	-0.70
**Importance of components:**			
Standard deviation	69.99	32.67	12.82
Proportion of Variance	0.80	0.17	0.03
Cumulative Proportion	0.80	0.97	1.00
