# Supplementary material for: Carotenoid coloration and health status of urban Eurasian kestrels (Falco tinnunculus)
Source: PLoS One. 2018 Feb 8;13(2):e0191956. doi: 10.1371/journal.pone.0191956 (PMC5805255; doi:10.1371/journal.pone.0191956)
Supplement: S1 Fig — We randomly selected one nestling per brood (n = 154 individuals, 154 broods, 91 different nest sites between 2010–2016) with an age range of six to 33 days. (PDF) [file pone.0191956.s002.pdf]

Supporting information:

"Carotenoid coloration and health status of urban Eurasian kestrels (*Falco tinnunculus*)"

Petra Sumasgutner, Marius Adrion, Anita Gamauf

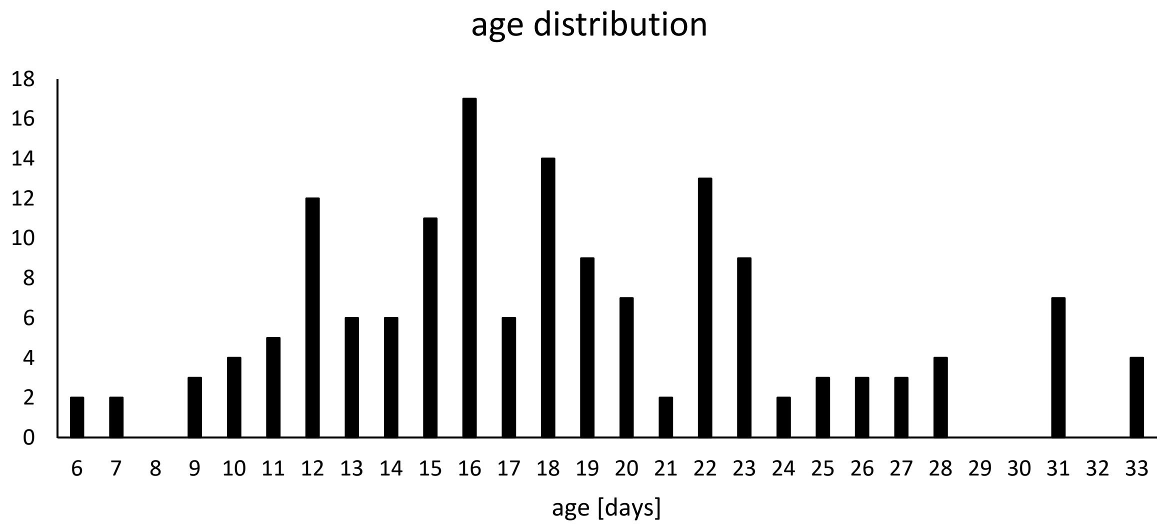

S1 Fig. Age distribution of kestrel nestlings considered for the analyses. We randomly selected one nestling per brood (n=154 individuals, 154 broods, 91 different nest sites between 2010-2016) with an age range of six to 33 days.
