# Supplementary material for: Carotenoid coloration and health status of urban Eurasian kestrels (Falco tinnunculus)
Source: PLoS One. 2018 Feb 8;13(2):e0191956. doi: 10.1371/journal.pone.0191956 (PMC5805255; doi:10.1371/journal.pone.0191956)
Supplement: S2 Fig — The model explains 51% of the variance in body mass (note: inward ticks on the x-axis show sample sizes). (PDF) [file pone.0191956.s003.pdf]

Supporting information:

"Carotenoid coloration and health status of urban Eurasian kestrels (*Falco tinnunculus*)"

Petra Sumasgutner, Marius Adrion, Anita Gamauf

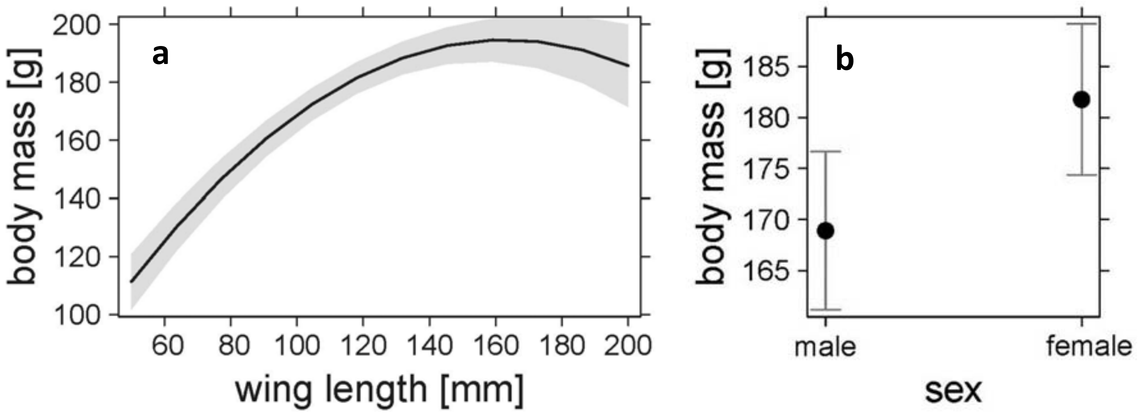

S2 Fig. Result of the best model of a GLMM on body mass of urban kestrel nestlings (see Table 1): Effects of (a) wing length, and (b) sex contribute significantly to the best model. The model explains 51% of the variance in body mass (note: inward ticks on the x-axis show sample sizes).
