# Supplementary material for: Carotenoid coloration and health status of urban Eurasian kestrels (Falco tinnunculus)
Source: PLoS One. 2018 Feb 8;13(2):e0191956. doi: 10.1371/journal.pone.0191956 (PMC5805255; doi:10.1371/journal.pone.0191956)
Supplement: S3 Fig — Effects of (a) age of nestlings, (b) nestling sex, (c) egg-laying date and urban gradient contribute significantly to the best model. The model explains 24% of the variance in face skin yellowness. (PDF) [file pone.0191956.s004.pdf]

Supporting information:

"Carotenoid coloration and health status of urban Eurasian kestrels (*Falco tinnunculus*)"

Petra Sumasgutner, Marius Adrion, Anita Gamauf

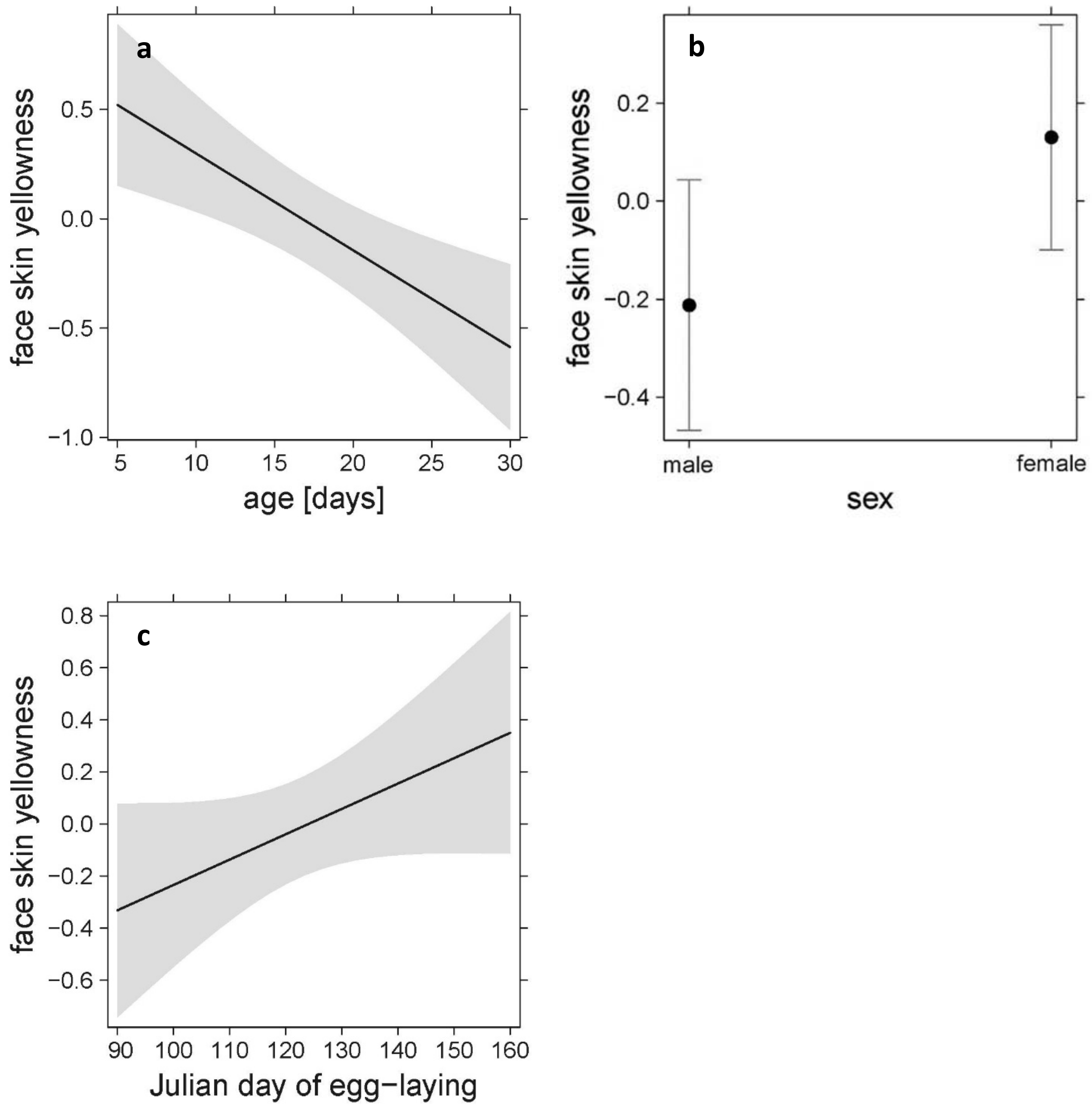

S3 Fig. Result of the best model of a GLMM on face skin yellowness of urban kestrel nestlings (see Table 1). Effects of (a) age of nestlings, (b) nestling sex, (c) egg-laying date and urban gradient contribute significantly to the best model. The model explains 24% of the variance in face skin yellowness.
